# Patient-Related Outcome Measures for Oculomotor Symptoms in the Cerebellar Ataxias: Insights from Non-Cerebellar Disorders

**DOI:** 10.1007/s12311-024-01656-3

**Published:** 2024-01-12

**Authors:** David J. Szmulewicz, Rocco Galli, Alexander A. Tarnutzer

**Affiliations:** 1 Balance Disorders and Ataxia Service, Royal Victoria Eye and Ear Hospital, Melbourne, VIC Australia; 2 The Bionics Institute, Melbourne, VIC Australia; 3 Faculty of Medicine, University of Zurich, Zurich, Switzerland; 4 Department of Neurology, Cantonal Hospital of Baden, Baden, Switzerland; 5 University of Melbourne AU, Melbourne, VIC Australia

**Keywords:** Quality of life, Vestibular, Oculomotor, Cerebellar ataxia, Rating scale, Patient related outcome measures, PROMs

## Abstract

In patients with cerebellar ataxia (CA), symptoms related to oculomotor dysfunction significantly affect quality of life (QoL). This study aimed to analyze the literature on patient-related outcome measures (PROMs) assessing QoL impacts of vestibular and cerebellar oculomotor abnormalities in patients with CA to identify the strengths and limitations of existing scales and highlight any areas of unmet need. A systematic review was conducted (Medline, Embase) of English-language original articles reporting on QoL measures in patients with vertigo, dizziness or CA. Pre-specified parameters were retrieved, including diseases studied, scales applied and conclusions drawn. Our search yielded 3671 articles of which 467 studies (*n* = 111,606 participants) were deemed relevant. The most frequently studied disease entities were (a) non-specific dizziness/gait imbalance (114 studies; 54,581 participants), (b) vestibular schwannomas (66; 15,360), and (c) vestibular disorders not further specified (66; 10,259). The Dizziness Handicap Inventory (DHI) was the most frequently used PROM to assess QoL (*n* = 91,851), followed by the Penn Acoustic Neuroma Quality-of-Life Scale (*n* = 12,027) and the Activities-Specific Balance Confidence Scale (*n* = 2’471). QoL-scores capturing symptoms related to oculomotor abnormalities in CA were rare, focused on visual impairments (e.g., National-Eye-Institute Visual Function Questionnaire, Oscillopsia Functional Impact, oscillopsia severity score) and were unvalidated. The DHI remains the most widely used and versatile scale for evaluating dizziness. A lack of well-established PROMs for assessing the impact of oculomotor-related symptoms on QoL in CA was noted, emphasizing the need for developing and validating a new QoL-score dedicated to the oculomotor domain for individuals with CA.

**Supplementary Information:**

The online version contains supplementary material available at 10.1007/s12311-024-01656-3.

## Introduction

Dizziness and vertigo are prevalent symptoms that can significantly diminish quality of life (QoL) [1, 2]. Oscillopsia, whether spontaneous or motion-induced, is less common but carries significant associated morbidity. Patients report a to-and-fro motion of their visual environment which results in reduced visual acuity that they often find disabling due to ocular instability [3], and has been shown to significantly affect an individual’s QoL [4]. Gait unsteadiness carries significant morbidity and adverse effect on patient’s QoL, and includes isolation and fear of falling [5]. It should be noted that these symptoms are not specific for any given pathology or anatomic system and as such, may be seen in vestibular and/or or cerebellar disease (or the combination of the two) [3]; and similarly for gait unsteadiness which may in part result from the oculomotor abnormalities seen in cerebellar and vestibular disease [1, 6]. In this paper, we consider the oculomotor symptoms referable to both cerebellar and vestibular impairment because (1) they result in a number of overlapping shared symptoms (e.g., vertigo, oscillopsia) and (2) there is an increasing recognition that vestibular dysfunction is a not uncommon component of an increasing number of CA phenotypes [7]. For the purposes of this paper, we are considering the symptoms of oculomotor abnormalities to include dizziness, vertigo, and oscillopsia. Oculomotor parameters, both of cerebellar and vestibular origin, are attractive targets of instrumented measurement [8, 9], however, in the current regulatory context, if they are to be utilized in treatment trials, would need to be accompanied by complimentary PROMs. Taken together, these points highlight the need for a validated PROM targeting this important domain of cerebellar dysfunction.

Cerebellar Ataxia (CA) is the motoric manifestation of cerebellar disease and may be summarized as impaired *coordination* which effects appendicular, balance and speech function amongst others [10, 11]. An important point of clarification is that a broad-based or ‘ataxic’ gait may broadly have three causes: cerebellar, vestibular and sensory dysfunction. Although a less common cause of imbalance than vestibular or somatosensory disease, CA often carries a very significant impact on an individual’s QoL [12, 13]. Established rating scales in CA such as the Scale for the Assessment and Rating of Ataxia (SARA) [14], the International Co-operative Ataxia Rating Scale (ICARS) [15] and the Brief Ataxia Rating scale (BARS) [16] have been designed to rate the range and severity of impairment in CA in a standardized way. More recently, the Scale for Ocular motor Disorders in Ataxia (SODA) was introduced, in order to allow a standardized rating of the extent of oculomotor deficits in cerebellar disorders [17]. However, these scales do not assess patient-related complaints or the influence of impairment on QoL.

Both the US Federal Drug and Food Administration (FDA) and the European Medicine’s Agency (EMA) [18–20] have stipulated the need for robust Patient Reported Outcomes (PROs) in the validation of new therapeutics. PROs refer to health or treatment outcomes reported directly by patients (without the interpretation of a clinician or another person). Patient Reported Outcome Measures (PROMs) are instruments that are used to measure the PROs, most often self-report questionnaires [21]. By way of example, the value of PROs has been emphasized in the field of oncology both for clinical trials and personalized cancer care [22]. For patients with CA, a validated general PROM for CA was recently created and validated, which addresses visual (blurred vision, double vision) and ‘vestibular’ (“spinning sensations, dizziness, vertigo or light-headedness”) complaints in single questions [23].

While various clinical scales have been developed to evaluate dizziness, vertigo and gait unsteadiness, it remains unclear to what extent these scores incorporated their impact on patients’ QoL and for which specific symptoms or diseases they are commonly used. We note that in a previous review of the literature (search period = 1991–2004) on questionnaires assessing the impact of vertigo and dizziness on patient’s QoL, the authors concluded that the review failed to identify any relevant and validated questionnaires [24].

To address these knowledge gaps, this study aims to analyze the current literature on PROMs [21] referable to symptoms of vestibular and cerebellar oculomotor abnormalities with a particular focus on those that incorporate QoL measures in patients with CA. This analysis aims to identify the limitations of existing scales and highlight areas where improvements and additions may be made. This systematic review is part of a broader initiative that aims to develop a novel scale for assessing QoL in patients with a CA who have oculomotor (cerebellar and/vestibular) symptoms.

## Methods

### Search Strategy

The search strategy was designed by a clinical investigator with relevant domain expertise in neurology (AAT). We searched MEDLINE and Embase for English-language articles, which met the following criteria: (1) defined the clinical syndromes or diagnoses examined (i.e., CA, dizziness, vertigo or gait imbalance), (2) enumerated the characteristics of QoL investigated, and (3) the latter must have addressed patient reported outcomes (PROs) and/or patient reported outcome measures (PROMs) on oculomotor and balance symptoms. Of note, we did not expressly search for specific hereditary ataxia syndromes (for example ‘spinocerebellar ataxia type 6’ or ‘spastic paraplegia type 7’), but focused on general search terms such as “cerebellar ataxia” and “gait imbalance”. We also performed a manual search of reference lists from eligible articles and contacted corresponding authors where necessary. We did not seek to identify research abstracts from meeting proceedings or unpublished studies.

### Abstract and Full-Text Reviews

Two independent investigators (RG and AAT) screened the identified studies by reviewing their titles and abstracts. Based on predefined exclusion criteria (see Appendix 1), the investigators decided whether to include or exclude studies. If both investigators recommended excluding a study, it was removed from consideration and no concordance of reason for exclusion was required. The studies that passed the initial screening were then subjected to a second screening. During this second stage, a full-text review was performed using similar exclusion criteria (see Appendix 1). However, concordance on inclusion or exclusion and the reason for exclusion was now required. Any discrepancies between the investigators assessments were resolved through discussion and consensus.

AAT completed a search of the selected article’s references to identify additional citations for inclusion. The same screening process was applied to these newly identified studies. The search was repeated iteratively until no further manuscripts were found for inclusion. A formal review protocol was not registered or posted. We calculated inter-rater agreement on full-text inclusion using Cohen’s kappa [25].

### Data Extraction, Synthesis and Analysis

For data extraction, pre-specified study parameters were retrieved. This included the type of study design used, the study setting, the number of patients and (if applicable) the number of control subjects studied, the study period and the disease entity/entities considered. Furthermore, the QoL score(s) applied were retrieved including whether reported differences in the scores obtained between distinct groups studied (patients vs. control subjects or different patient groups) reached statistical significance (*p* < 0.05) or not. We also obtained information – if provided by the authors - on the usefulness of the score(s) reported on the monitoring of disease progression or treatment response. This information was retrieved from the discussion / conclusions section of these manuscripts and reflected the authors own assessment. Statistical analysis was descriptive. Being a systematic review, no ethical approval was necessary for this study.

### Data Availability

Source data used for this systematic review will be made available to others upon request to the corresponding author.

## Results

### Search Results

Our search identified 3671 citations, of which 2577 (70.2%) were excluded at the abstract level. We examined 1094 (29.8%) manuscripts at the full-text level. After initial screening, there were a total of 57 disagreements about study inclusion/exclusion amongst the two reviewers (RG and AAT, kappa = 0.89). These differences were resolved by discussion. Overall, initial agreement on the reason for exclusion was 58.9%. We demanded concordance on the reason for full-text exclusion and resolved differences by discussion.

**Fig. 1:**
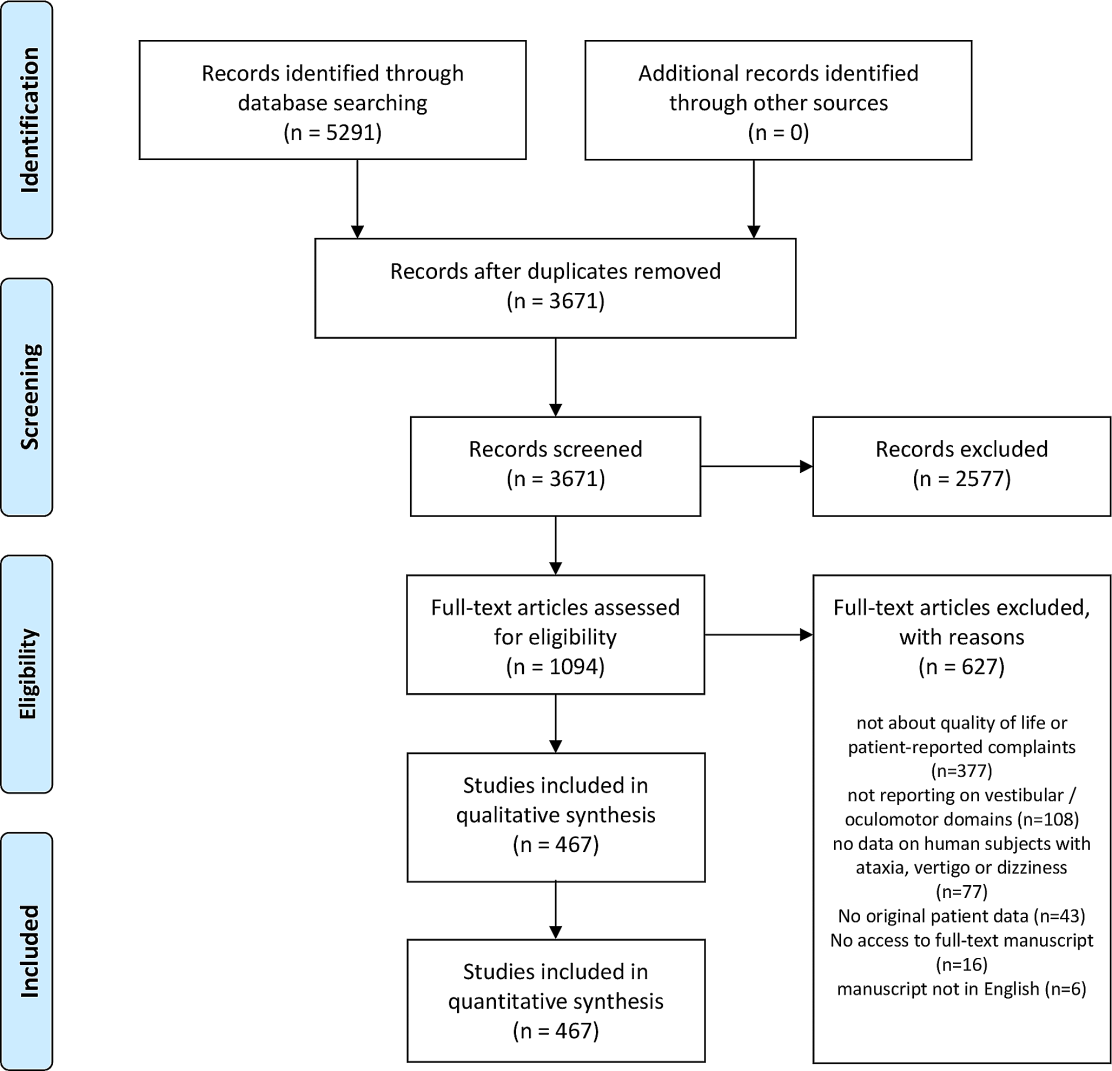
PRISMA flow chart

At the end of our full-text review, 627 (57.3%) articles were excluded and 467 (42.7%) articles were considered eligible. These eligible studies represented 12.7% of the total 3671 articles initially identified. Among the full-text manuscripts excluded (17.1%), the distribution of reasons for exclusion was as follows: 60.1% did not address QoL or patient-reported symptoms; 12.3% did not include relevant human derived data; 6.9% did not contain original data; 17.2% were not reporting on PROs / PROMs in the oculomotor domain; in 2.6% a full-text manuscript could not be retrieved, and 1.0% were not available in English (Fig. 1 [PRISMA flow chart]).

## Study Characteristics

Of the 467 studies included in the final selection, 327 had a prospective design (70.0%), 134 had a retrospective design (28.7%), and 6 had a hybrid design (1.3%). The study types included 226 cross-sectional studies with only one measurement (48.4%), 181 interventional studies with measurements taken at baseline, during the intervention, and then again afterward (38.8%), and 60 observational studies with several measurements taken over time (12.8%).

### Most Common Symptoms and Disorders Studied

We identified a broad range of different leading symptoms and disorders reported in the selected studies. The five most frequently studied categories, which also made up the majority of participants across all the studies were: patients with dizziness or gait imbalance not further specified (i.e., with regards to the underlying cause or clinical presentation) in a total of 114 studies (53,769 patients); vestibular schwannomas (acoustic neuroma), with 66 studies (15,220 patients); vestibular disorders not further specified, 66 studies (8,476 patients); benign paroxysmal positional vertigo (BPPV), 30 studies (2,183 patients); and Menière’s disease, 28 studies (2,109 patients). It is worth noting that concussion was also a significant category, with 16,400 patients across 21 studies, indicating studies with larger sample sizes. In total, there were 68 (out of 467, 14.6%) studies that included control subjects. The number of control subjects included across all studies was 4,483, representing 4.0% of the total number of participants in the studies included in this systematic review. For the complete list of leading symptoms/disorders included the number of studies they were found in, and the number of patients, see Table 1.

**Table 1:** Distribution of presenting symptoms, disorders and diseases

Presenting symptom/disease diagnosed	Studies (*n*)	Patients (*n*)
***Symptom-based inclusion***		
Ataxia	3	76
Concussion	21	16’400
Dizziness or gait imbalance	114	53’769
Visual vertigo	2	143
***Inclusion based on unspecified disorders***		
Patients with a cochlear implant	9	535
«Vestibular» disorders (not further specified)	66	8’476
***Inclusion based on specific diseases***		
*Peripheral-vestibular disorders*		
Bilateral vestibulopathy	14	448
BPPV	30	2’183
Menière’s disease	28	2’109
Neurofibromatosis II	3	163
SSCDS	6	170
SSNHL	2	255
UPV		
Acute UPV	17	918
Chronic UPV	5	199
UPV (not further specified)	22	971
Vestibular schwannoma	66	15’220
Vestibular migraine		
Somatoform dizziness		
Parkinson’s disease	5	106
*Neuroinflammatory disorders*		
Multiple sclerosis	3	179
*Hereditary ataxias*	4	86
Various, specific disorders*	6	1’111
*Miscellaneous§*	10	597
**Total**	**467**	**106’723**

* This included 1 study with patients with vestibular migraine or BPPV (176 patients), 1 study with patients with either vestibular migraine or persistent postural-perceptual dizziness (60 patients), 1 study with patients with vestibular migraine or Mal de dèbarquement syndrome (62 patients), 1 study with patients with vestibular migraine or Menière’s disease (761 patients), 1 study with patients with BPPV or Menière’s disease (12 patients), and 1 study with patients with vestibular schwannoma or persistent whiplash symptoms (40 patients)

§ This includes 1 study each with “central vestibular disorders” not further specified (72 patients), cervicogenic dizziness (20 patients), glomus jugular tumors (30 patients), Mal de débarquement (27 patients), MELAS (8 patients), otosclerosis (33 patients), patients receiving (non-vestibular) surgery (287 patients), patients with falls (30 patients), post COVID19 infection patients (50 patients), and patients status post whiplash (20 patients)

Abbreviations of disorders and diseases: BBPV, Benign paroxysmal positional vertigo; CI, Cochlear Implant; PPPD, Persistent Postural Perceptual Dizziness; MELAS, Mitochondrial encephalomyopathy, lactic acidosis and stroke-like episodes; SSCDC, Superior Semi-Circular Canal Dehiscence Syndrome; SSNHL, Sudden Sensorineural Hearing Loss; UPV, Unilateral Peripheral Vestibulopathy

### Distribution of the Quality-of-Life Scores

Of the 467 studies and 111,606 participants included in this systematic review, the Dizziness Handicap Score (DHI) was utilized in 374 studies (80.1%, 91,851 participants), covering all categories of leading symptoms and disorders identified in this review, except neurofibromatosis 2 [26]. The Penn Acoustic Neuroma QoL Scale (PANQOL/PANQL Scale) followed, with 35 studies (7.5%, 12,027 participants), and was exclusively used in studies reporting on patients with vestibular schwannomas [27]. The Activities-Specific Balance Confidence Scale (ABC Scale) was found in 29 studies (6.2%, 2,471 participants), and primarily applied to patients with dizziness or gait unsteadiness in vestibular disorders such as unilateral peripheral vestibulopathy and in patients that have suffered from mild traumatic brain injury (mTBI, also referred to as concussion) [28]. The Vestibular Disorders Activities of Daily Living Scale (VADL Scale) was utilized in 25 studies (5.4%, 1,919 participants), and was used across a variety of conditions, principally in patients who reported dizziness or gait unsteadiness in vestibular disorders [29]. The University of California Los Angeles Dizziness Questionnaire (UCLA-DQ) was used in 13 studies (1’571 participants), chiefly in patients with dizziness or gait unsteadiness [30]. The Vertigo Handicap Questionnaire (VHQ) was found in 10 studies (1’226 participants), predominantly in studies reporting on patients with vestibular disorders and functional vestibular dizziness, including Persistent Postural Perceptual Dizziness (PPPD) [31]. The Vestibular Activities and Participation Measure (VAP) was found in four studies (671 participants) and was used in studies reporting on dizziness or gait unsteadiness in patients with vestibular disorders including BPPV, and functional dizziness such as PPPD [32]. The Menière’s Disease Outcome Questionnaire (MDOQ) was used in four studies with a total of 191 participants, all of whom suffered from Menière’s disease [33]. Likewise, the Migraine Disability Assessment scale (MIDAS) was identified in four studies (204 participants) to patients with confirmed vestibular migraine. The Glasgow Benefit Inventory (GBI) was applied in two studies with a total of 254 participants with vestibular schwannoma [34]. For a complete list of the scores analyzed and their distribution by frequency and category of disorders, see Table 2.

**Table 2:** QoL scores and their distribution

	Qol scores used (studies; participants)													
Presenting symptom/diagnosis made	**DHI**	**PANQL**	**ABC**	**VADL**	**UCLA**	**VHQ**	**VAP**	**MDOQ**	**GBI**	**NEI-VFQ**	**OFI**	**BDC**	**OSQ**	**Other scores**
***Symptom-based inclusion***														
Ataxia	1; 2	0; 0	0; 0	0; 0	0; 0	0; 0	0; 0	0; 0	0; 0	0; 0	0; 0	0; 0	0; 0	2; 74
Concussion	20; 16’617	0; 0	5; 176	0; 0	0; 0	0; 0	0; 0	0; 0	0; 0	0; 0	0; 0	0; 0	0; 0	6; 374
Dizziness/gait imbalance	99; 52’275	0; 0	5; 545	5; 638	8; 1’293	1; 100	1; 53	0; 0	0; 0	0;0	0; 0	0; 0	0; 0	15; 2’095
Visual vertigo	2; 168	0; 0	1; 103	0; 0	0; 0	0; 0	0; 0	0; 0	0; 0	0; 0	0; 0	0; 0	0; 0	0; 0
***Inclusion based on unspecified disorders***														
Patients with CI	9; 535	0; 0	0; 0	0; 0	0; 0	0; 0	0; 0	0; 0	0; 0	0; 0	0; 0	0; 0	0; 0	0; 0
«Vestibular» disorders (not further specified)	56; 7’675	0; 0	5; 319	10; 578	0; 0	4; 831	1; 58	0; 0	0; 0	0; 0	0; 0	0; 0	0; 0	4; 1’652
***Inclusion based on specific diseases***														
*Peripheral-vestibular disorders*														
Bilateral vestibulopathy	12; 371	0; 0	0; 0	0; 0	0; 0	0; 0	0; 0	0; 0	0; 0	0; 0	1; 69	1; 12	1; 26	3; 113
BPPV	26; 1’994	0; 0	0; 0	2; 158	0; 0	0; 0	1; 90	0; 0	0; 0	0; 0	0; 0	0; 0	0; 0	3; 168
Menière’s disease	22; 1’232	0; 0	0; 0	1; 221	1; 103	0; 0	0; 0	4; 191	0; 0	0; 0	0; 0	0; 0	0; 0	7; 1’706
Neurofibromatosis II	0; 0	0; 0	0; 0	0; 0	0; 0	0; 0	0; 0	0; 0	0; 0	0; 0	0; 0	0; 0	0; 0	3; 183
SSCDS	6; 170	0; 0	0; 0	0; 0	0; 0	0; 0	0; 0	0; 0	0; 0	0; 0	0; 0	0; 0	0; 0	0; 0
SSNHL	2; 255	0; 0	0; 0	0; 0	0; 0	0; 0	0; 0	0; 0	0; 0	0; 0	0; 0	0; 0	0; 0	0; 0
UPV														
Acute UPV	14; 869	0; 0	2; 74	1; 35	1; 65	0; 0	0; 0	0; 0	0; 0	0; 0	0; 0	0; 0	0; 0	4; 139
Chronic UPV	4; 212	0; 0	0; 0	1; 75	0; 0	0; 0	0; 0	0; 0	0; 0	0; 0	0; 0	0; 0	0; 0	0; 0
UPV (not further specified)	20; 1’132	0; 0	6; 535	1; 70	2; 88	0; 0	0; 0	0; 0	0; 0	0; 0	0; 0	0; 0	0; 0	3; 235
Vestibular schwannoma	29; 3’527	35; 12’2027	1; 539	0; 0	1; 22	2; 123	0; 0	0; 0	2; 254	0; 0	0; 0	0; 0	0; 0	3; 393
Vestibular migraine	17; 1’652	0; 0	2; 124	2; 98	0; 0	0; 0	0; 0	0; 0	0; 0	0; 0	0; 0	0; 0	0; 0	3; 205
Somatoform dizziness	12; 1’135	0; 0	0; 0	0; 0	0; 0	3; 172	1; 470	0; 0	0; 0	0; 0	0; 0	0; 0	0; 0	4; 470
Parkinson’s disease	4; 95	0; 0	1; 16	1; 16	0; 0	0; 0	0; 0	0; 0	0; 0	0; 0	0; 0	0; 0	0; 0	1; 52
*Neuro-inflammatory disorders*														
Multiple sclerosis	3; 179	0; 0	1; 40	0; 0	0; 0	0; 0	0; 0	0; 0	0; 0	0; 0	0; 0	0; 0	0; 0	1; 40
*Hereditary ataxias*	1; 28	0; 0	0; 0	0; 0	0; 0	0; 0	0; 0	0; 0	0; 0	2; 53	0; 0	0; 0	0; 0	3; 115
Various, specific disorders*	6; 1’161	0; 0	0; 0	0; 0	0; 0	0; 0	0; 0	0; 0	0; 0	0; 0	0; 0	0; 0	0; 0	0; 0
*Miscellaneous§*	9; 567	0; 0	0; 0	1; 30	0; 0	0; 0	0; 0	0; 0	0; 0	0; 0	0; 0	0; 0	0; 0	0; 0
***Total***	**374; 91’851**	**35; 12’027**	**29; 2’471**	**25; 1’919**	**13; 1’571**	**10; 1’226**	**4; 671**	**4; 191**	**2; 254**	**2; 53**	**1; 69**	**1; 12**	**1; 26**	**62; 8’014**

* This included 1 study with patients with vestibular migraine or BPPV (176 patients), 1 study with patients with either vestibular migraine or persistent postural-perceptual dizziness (60 patients), 1 study with patients with vestibular migraine or Mal de dèbarquement syndrome (62 patients), 1 study with patients with vestibular migraine or Menière’s disease (761 patients), 1 study with patients with BPPV or Menière’s disease (12 patients), and 1 study with patients with vestibular schwannoma or persistent whiplash symptoms (40 patients)

§ This includes one study each with central vestibular disorders not further specified (72 patients), cervicogenic dizziness (20 patients), glomus jugular tumors (30 patients), Mal de débarquement (27 patients), MELAS (8 patients), otosclerosis (33 patients), patients receiving (non-vestibular) surgery (287 patients), patients with falls (30 patients), post COVID19 infection patients (50 patients), and patients status post whiplash (40 patients)

Abbreviations of QoL scores: ABC, Activities-Specific Balance Confidence Scale; BDC, Balance Disorder Checklist; DHI, Dizziness Handicap Inventory; GBI, Glasgow Benefit Inventory; MDOQ, Mani?re?s Disease Outcome Questionnaire; NEI-VFQ, National Eye Institute Visual Function Questionnaire; OFI, Oscillopsia Functional Im-pact Scale; OSQ, Oscillopsia Severity Questionnaire; PANQOL/PANQL Scale, Penn Acoustic Neuroma Quality of Life Scale; UCLA-DQ, University of California Los Angeles Dizziness Questionnaire; VADL Scale, Vestibular Disorders Activities of Daily Living Scale; VAP, Vestibular Activities and Participation Measure; VHQ, Verti-go Handicap Questionnaire

Abbreviations of disorders and diseases: BBPV, Benign paroxysmal positional vertigo; CI, Cochlear Implant; PPPD, Persistent Postural Perceptual Dizziness; SSCDC, Superior Semi-Circular Canal Dehiscence Syndrome; SSNHL, Sudden Sensorineural Hearing Loss; UPV, Unilateral Peripheral Vestibulopathy.

We identified several scores that restricted QoL to vision-related impairment in patients with imbalance, vertigo, dizziness and/or ataxia. This included the National Eye Institute Visual Function Questionnaire (NEI-VFQ) with several extensions such as the NOS (neuro-ophthalmologic extension) or the NEI-VFQ-39 [35–37] and the Visual Evaluation Protocol (VISATAX) [38]. These tools were used in studies of patients with CAs (for details see next section). The Oscillopsia Functional Impact (OFI) Scale, Oscillopsia Score (OS) and Oscillopsia Severity Questionnaire (OSQ) have been applied in patients with bilateral vestibulopathy (OFI scale [*n* = 69 patients [39]], OS [*n* = 12 patients [40] and *n* = 12 patients [41]], and OSQ [*n* = 26 patients [42] and *n* = 39 patients [4]]). Additionally, the OS was used in patients with unilateral peripheral vestibulopathy (*n* = 18 patients [41]).

### Quality-of-Life Scores in Cerebellar Ataxias

The NEI-VFQ-39 [43] was used in three studies with a total of 56 patients with various spinocerebellar ataxias [36, 37] and Friedreich ataxia [35]. In one study [35], it was combined with the VF-14 (visual function 14) score, which was designed to assess visual functional impairment in patients with cataracts [44]. For a complete and in-depth overview of the studies dealing with hereditary ataxia, see Table 3.

**Table 3:** Hereditary ataxias distribution

	SCA1	SCA2	SCA3	SCA4	SCA5	SCA6	SCA8	SCA10	SCA14	SCA ND	FRDA	CAUE	QoL scores used
Alexandre et al. 2013 [38]	6		6								9		VISATAX Q.
Fahey et al. 2008 [35]											20		VF14 and NEI-VFQ-39
Ihl et al. 2020 [37]									17				NEI-VFQ-39 and NOS
Joyce et al. 2022 [53]	2	5	6		1	10	2			10		15	Semi-structured Interview
Kedar et al. 2015 [36]	3		11			5							NEI-VFQ 25 and NOS
Santos et al. 2017 [45]		2	5	1				5		7	8		DHI
**Total**	**11**	**7**	**28**	**1**	**1**	**15**	**2**	**5**	**17**	**17**	**37**	**15**	

Abbreviations of QoL scores used: DHI, Dizziness Handicap Inventory; NEI-VFQ, National Eye Institute Visual Function Questionnaire; NOS, neuro-ophthalmologic extension; Q., Questionnaire; VF14, Visual Function 14

Abbreviations of disorders and diseases: CAUE, Cerebellar Ataxia Undefined Aetiology; FRDA, Friedreich’s ataxia; ND, not defined; SCA, Spinocerebellar Ataxia

### The Perceived Value of Quality-of-Life Scores in the Included Studies

In all of the included studies, we evaluated whether the score(s) reported were significantly different between the patient and control groups or between different patient groups. We focused on the most frequently used scores, specifically, the DHI, VADL, PANQL, ABC, VHQ and UCLA-DQ. A significant difference in the QoL scores between the distinct groups compared was identified in the vast majority of the studies (ranging from 77.1 to 100%). Focusing on those studies monitoring disease progression or treatment response (*n* = 241), the score(s) used were considered helpful by the authors in 44.4–100% of the studies, with the lowest values for the PANQL (44.4%) and the VHQ (80%) (see Table 4 for details). Note that a single study of patients with several spinocerebellar ataxias reported on treatment response (using virtual reality-based balance rehabilitation) [45] and demonstrating significant improvement of QoL in the DHI.

**Table 4:** The perceived value of QoL scores*

Score	Score significantly different between patients and controls or between different patient groups (%)	QoL-score considered helpful in monitoring treatment response or progression by the authors (%)
DHI	318/374 (85.0%)	177/207 (85.5%)
PANQL	27/35 (77.1%)	4/9 (44.4%)
ABC	28/29 (96.6%)	15/15 (100%)
VADL	22/25 (88.0%)	14/17 (82.4%)
UCLA-DQ	13/13 (100%)	7/7 (100%)
VHQ	9/10 (90.0%)	4/5 (80.0%)

* Note that scores that were reported in less than 10 studies were not included

Abbreviations of QoL scores: ABC, Activities-Specific Balance Confidence Scale; DHI, Dizziness Handicap Inventory; PANQOL/PANQL Scale, Penn Acoustic Neuroma Quality of Life Scale; UCLA-DQ, University of California Los Angeles Dizziness Questionnaire; VADL Scale, Vestibular Disorders Activities of Daily Living Scale; VHQ, Vertigo Handicap Questionnaire

## Discussion

Focusing on patient related outcome measures (PROMs) in patients with vestibular and cerebellar disorders, this systematic review identified various scores available for the assessment of quality of life (QoL) related to vestibular and oculomotor complaints. Specifically, the DHI was found to be the most widely employed scale for evaluating vertigo or dizziness, surpassing the second-most used scale (PANQL) by a factor of 10, and exceeding the sum of the next five most commonly utilized scales by a factor of 3. Overall, there was significant heterogeneity in the patient populations studied and often poorly defined symptom-oriented diagnoses such as “unspecific dizziness or gait imbalance” (50.4% [53,769 of 106,723 patients]) or “vestibular disorders not further specified“ (7.9% [8’476 of 106’723 patients]) were used. Importantly, in the majority of studies the most commonly applied scales facilitated a statistically significant distinction between patient and control cohorts or different patient cohorts studied (77–100% depending on the scale used). However, the perceived value of self-reported metrics in monitoring disease progression or treatment response was more variable, considered helpful in 44 to 100% of studies (depending on the scale used, see Table 4 for details). In the following we will discuss the strengths and limitations of currently used QoL scores addressing oculomotor and/or vestibular complaints and put a special focus on their (future) use in patients with cerebellar ataxia (CA).

### A Critical Review of the Most Commonly Used QoL Measures for Patients with Dizziness, Vertigo or Cerebellar Ataxia

The DHI aims to grade the subjective impact of dizziness on QoL [24]. It was developed to be employed in the context of vestibular disease and whilst it has good measurement properties and has been applied over a broad range of symptoms and diseases, a recent systematic review has highlighted some limitations. Specifically, in the studies identified in this systematic review evidence pertaining to the DHI’s content validity was either lacking or limited and of low quality and there was very limited evidence to support sufficient reliability of the DHI total score [46]. There is a single item in the DHI that may potentially relate to motion-induced oscillopsia, although it is rather non-specific (“Because of your problem, do you have difficulty reading?”). While the DHI is validated for symptoms of vestibular disease, it is not validated for patients with CA and in the research context has been rarely applied to CA patients [45].

This systematic review found that the VHQ and the UCLA-DQ were applied less frequently than the DHI for QoL assessment in dizzy patients. For the UCLA-DQ some studies reported good validity and reliability, while others did not [31, 47]. Other scales, such as the ABC, VAP, and VADL focus on activities of daily living that may be affected by dizziness such as mobility, fear of falling (ABC) and activities of daily living (VADL), but had limited (VADL) or absent (ABC) specificity to vertigo [24]. Several scales focused on visual symptoms, such as the NEI-VFQ (and its extensions), OFI, OS and OSQ. While vision is a crucial factor in evaluating the QoL of a patient experiencing dizziness and/or ataxia, it is not the sole determining factor. Thus, it is essential to consider the specialization of these scoring systems.

### Discriminatory Value of QoL Scores Used in Dizzy and/or Ataxic Patients, and Their Role in Monitoring Disease Progression and Treatment Response

In a majority of studies included, the instruments applied demonstrated statistically significant differences between patients and controls, or between patient groups. This substantiates the role of the relevant tools in identifying the impact of vertigo, dizziness, or oculomotor symptoms on patient’s QoL. For the scales used in 10 or more studies (DHI, VADL, PANQL, ABC, VHQ and UCLA-DQ), a significant difference between patients and controls was reported in 77–100% of studies (depending on the tool used), with the highest values (> 95%) being for the UCLA-DQ and the ABC.

In this review, we gathered information on the authors’ perceived value of the scale(s) as applied in monitoring disease progression or treatment response. Focusing on longitudinal observational or interventional studies, the perceived value of the scores varied substantially. For the DHI, UCLA-DQ, VADL, VHQ and ABC 80% or greater of the studies reported that the scale(s) utilized were clinically valuable, however, the PANQL was only considered to be of benefit in a substantially lower fraction (44.4% of studies). Potential reasons for the lower rate of perceived utility of the PANQL by study authors include the design of the PANQL questionnaire, being less sensitive in detecting changes in QoL over time, and the natural course of disease in vestibular schwannoma (which is generally stable or slowly progressive such that no significant differences in response to various treatments (surgery vs. radiation therapy vs. expectant management) are seen).

### The Value of Specific QoL Scores in Patients with Cerebellar Ataxia

With a special focus on developing a new QoL score focusing on the symptoms of oculomotor impairment in cerebellar and vestibular impairment in patients with CA (hereditary, sporadic, or acquired), we have compiled a subjective list of scores in order of perceived potential, which may serve as a basis for developing a new QoL score related to unsteadiness, oculomotor and vestibular involvement in CA patients.

### NEI-VFQ-39 and NOS Extension of NEI-VFQ-25

The NEI-VFQ-39, a 39-item questionnaire, which is based on the VFQ-25 questionnaire [43], but includes an additional 14 items from the original 51-item version of the VFQ [48], is of particular interest given its application in three of the articles that we identified which reported on patients with cerebellar ataxias [35–37]. However, both the NEI-VFQ-39 and the NEI-VFQ-25 focus solely on the impact of vision-related symptoms on patients’ QoL and therefore neglect to consider other potential symptoms commonly experienced by those with CA. The NEI-VFQ-25 was designed to address QoL in patients with chronic ophthalmological diseases such as cataracts, glaucoma, age-related macular degeneration, diabetic retinopathy, or CMV retinitis. It is possible that a more comprehensive PRO for use in patients with CA could combine this with other scales such as the SODA [17]. It is also important to note that the NEI-VFQ-39 scoring system was not specifically designed for addressing visual or oculomotor symptoms typically seen in CA but rather for a wide range of neuro-ophthalmologic disorders. Nevertheless, it serves as a well-established questionnaire for assessing critical aspects of vision-related QoL [48].

A 10-item neuro-ophthalmologic extension (NOS) of the NEI-VFQ-25 questionnaire has been utilized in order to increase the questionnaire’s capacity to capture self-reported visual dysfunction in patients with neuro-ophthalmologic disorders [49]. This study included patients with optic neuritis, multiple sclerosis, idiopathic intracranial hypertension, ischemic optic neuropathy, stroke, ocular myasthenia gravis, ocular motor palsies, and thyroid eye disease. The NOS supplement demonstrated a capacity to capture self-reported visual dysfunction beyond that of the NEI-VFQ-25 alone [49]. Whilst appropriate degrees of internal consistency reliability were demonstrated, the patient cohort was of very varied pathology and it is likely, that few or no patients had certain oculomotor abnormalities commonly seen in CA such as spontaneous or gaze-evoked nystagmus. Additionally, these patients likely had intact vestibular function. While this scale includes relevant items such as blurred vision and double vision, there is an absence of items which could identify oscillopsia.

In patients with Friedreich Ataxia (FA), significant Pearson correlations (p≤0.01) between subitems of the NEI-VFQ-39 which relate to general vision and near activities, and the SLCLC (Sloan Low Contrast Letter Chart) were reported and correlated with decreased visual QoL. However, no such correlations were observed between the NEI-VFQ-39 and the Friedreich Ataxia Rating Scale (FARS) or commonly occurring oculomotor abnormalities in FA measures including angular Vestibulo-Ocular Reflex (aVOR) gain, the presence of saccadic eye pursuit or saccadic intrusions [35].

In a study reporting on visual system involvement in spinocerebellar ataxia (SCA) type 14, the patients rated their vision-related QoL in the NEI-VFQ (combining the original VFQ-25, the 14 questions from the appendix and the 10-item NOS proposed by Raphael and colleagues [49]) significantly worse than control subjects [37]. Importantly, only 3 out of 12 patients included in this study had oscillopsia.

In a study reporting on 19 SCA patients (11 SCA 3, 3 SCA 1 and 5 SCA 6), scores for the NEI-VFQ-25 and the NOS extension were significantly reduced in the patients compared to a reference population [36]. The authors concluded that the results of this study supported screening of SCA patients for visual disability. Impaired ocular stability on lateral gaze (“end-gaze nystagmus”) was reported in five out of 12 patients studied, however, none of these patients reported oscillopsia. Furthermore, no information was provided about vestibular properties, e.g., the integrity of the aVOR.

#### Oscillopsia Functional Impact Scale and Oscillopsia Severity Questionnaire

The Oscillopsia Functional Impact Scale (OFI) questionnaire exhibits similar limitations as the NEI-VFQ-39, given its exclusive focus on oscillopsia. While it is well-suited for evaluating the impact of oscillopsia on the patients’ QoL, other relevant balance-related symptoms of CA may be overlooked. Additionally, the authors of the questionnaire acknowledged the length of this instrument may pose a challenge and suggested the development of a more concise and practical version. Perhaps most importantly, the questionnaire’s test-retest reliability has yet to be evaluated [39].

The oscillopsia severity questionnaire (OSQ) was initially proposed for quantifying the oscillopsia severity in patients with bilateral vestibulopathy [4]. These authors found that the OSQ strongly correlated with the DHI and this may be viewed as support for its use in the assessment of oscillopsia severity in patients with bilateral vestibulopathy [4]. As the OSQ focuses on the severity of the symptom during ADLs, it does not specifically assess its impact on QoL. While the severity of oscillopsia may provide an indication of its impact on the patient’s QoL, this measure does not directly inquire about the patient’s ability to navigate in space independently and clearly perceive objects, especially during head movements.

Similarly, an oscillopsia questionnaire (OS) was developed as part of the BDC and was correlated to retinal slip in patients with bilateral vestibulopathy who presented with oscillopsia [40]. This oscillopsia questionnaire addresses both the severity of complaints (from “no difficulty” to “cannot do”) and their impact on QoL. It also specifically interrogates for oscillopsia triggered by head movements, and provides an oscillopsia score. The authors demonstrated that in these patients greater oscillopsia handicap scores were significantly correlated with a greater external locus of control, i.e., the perception of having little control over one’s health [40]. More recently, an Italian (unvalidated) version of this questionnaire was used in patients with either unilateral or bilateral vestibular hypofunction [41]. These authors demonstrated that functional vestibular testing (dynamic visual acuity, DVA) and the OS were highly correlated.

#### Visual Evaluation Protocol (VISATAX)

In a single study, patients with SCA 1, SCA 3, or FA were required to fill out a subjective Visual Evaluation Protocol (VISATAX) [38]. The authors demonstrated that the VISITAX score was increased in the majority of the patients studied (59%), being the highest in the SCA 3 group. Noteworthy, the VISATAX score was not correlated to any oculomotor parameter retrieved and no detailed information was provided on the prevalence of oscillopsia in this patient cohort.

#### Dizziness Handicap Inventory (DHI)

While the versatility of the DHI enables its use across various disorders [50], we hold the view that its scope is rather superficial and does not provide precise insight into the specific symptoms that significantly impact the CA patient. A single item (question F7 – “Because of your problem, do you have difficulty reading”?) addresses visual impairment, but not focusing on its occurrence while moving the head – i.e., not addressing typical situations that may trigger oscillopsia. As a matter of fact, in our review, it was only utilized in two studies involving a total of thirty patients who presented with CA and no validation of the DHI in CA was provided [45, 51].

#### Other Scores that have not Been Applied to CA Patients

The Glasgow Benefit Inventory (GBI) was designed to measure the improvement experienced by a patient following surgical or medical otolaryngology interventions for conditions including vestibular schwannoma. Therefore, it may be appropriate to use it if a CA patient undergoes an intervention that potentially improves (ataxic) gait, oscillopsia or deficits in the aVOR. However, outside of such circumstances, it does not appear to be a promising tool for the self-assessment of ataxia patients.

The Balance Disorder Checklist (BDC) presents an attempt to evaluate two crucial aspects of vestibular disease: balance impairment and specific visual symptoms, i.e., oscillopsia, while also considering the severity of the symptom and their impact on activities of daily living (ADLs). However, one potential limitation of the scale lies in its scoring method, which appears to vary between questions (requiring participants to indicate the dynamics of symptom onset for certain complaints (“spinning sensation” and “wobbling, jumping, or blurring of vision”) but not for others), possibly leading to increased complexity in its application and interpretation. Importantly, the reliability and internal consistency of this scale were assessed in a single study involving 12 patients with isolated peripheral bilateral vestibulopathy only, thereby necessitating further research to establish the scale’s robustness and validity in other diseases including CA [40]. Other scores identified in this systematic review such as the UCLA-DQ, the VHQ, the ABC scale, the Vestibular Activities and Participation Measure (VAP) and the VADL have not been used in patients with CA. Thus, their potential value for CA patients remains unclear and subject to further studies.

Overall, considering the available range of potential instruments for evaluating CA patients, it becomes evident that while several promising options exist, there is a notable absence of specific, validated instruments that comprehensively assesses the QoL of this particular patient group across various domains.

### Key Items and Sub-Items in the Identified Scales Which Apply to Patients with Oscillopsia and/or CA

Scales with items of potential relevance in measuring PROs relating to QoL in individuals with CA scores include the oscillopsia score (OS) [40], the VISATAX score [38], the Oscillopsia Severity Questionnaire (OSQ) [4], the Oscillopsia Functional Impact Scale (OFI) [39], the Neuro-Ophthalmologic extension (NOS) of the NEI-VFQ-25 questionnaire [49] and the NEI-VFQ-39 questionnaire [48]. These scales have been hitherto applied to various patient populations including those with unilateral [41] or bilateral vestibular deficits [4, 40, 41], Friedreich Ataxia [35, 38], various SCAs (1, 3, 6, 14) [36–38], and neuroinflammatory diseases such as optic neuritis and multiple sclerosis [49]. While some scales such as the OS [40], OSQ [4] and the OFI [39] focus on oscillopsia, others address visual disturbances in a broader sense (including photophobia, difficulties focusing on objects, ptosis and diplopia) as e.g. the VISATAX [38], the NEI-VFQ-39 [43, 48] and the NOS of the NEI-VFQ-25 questionnaire [49]. Whereas all scores identified used Likert scales, they provided different types of questions, including agreement questions (NOS extension of the NEI-VFQ-25, NEI-VFQ-39), symptom intensity questions (VISATAX), questions focusing on difficulty handling certain situations (OS, NOS of the NEI-VFQ-25, NEI-VFQ-39) and frequency questions (OFI, OSQ, NOS of the NEI-VFQ-25, NEI-VFQ-39). Thus, these scales covered at least partially distinct aspects of PROMs related to oculomotor deficits and their impact of QoL. However, none of these scores had been specifically developed or validated in patients with CA (covering aspects such as internal consistency, content validity, test-retest reliability, discriminant ability, and responsiveness). Thus, these instruments are unable to capture the entire spectrum of oculomotor and balance symptoms typically observed in patients with CA, but rather, may identify a limited number of PROs. Therefore, developing a tool that specifically assesses the impact of oculomotor and balance deficits on QoL in patients with CA is strongly recommended. Traditionally employed scales in CA such as the SARA [14], ICARS [15] or the BARS [16] either do not address oculomotor symptoms (SARA), or do not address their impact on QoL (BARS, ICARS, SODA). However, the SODA [17] and the PROM ataxia [23] may serve as a starting point together with single items from scales designed to identify the impact of oscillopsia and gait imbalance on QoL in other diseases including bilateral vestibulopathy.

### Limitations

While our systematic review has provided valuable insights into the utilization of QoL scores, there are several limitations that must be considered. Firstly, we encountered several studies that utilized ambiguous terminologies in their account of the primary symptoms and disease(s) studied. This required us to pool studies in more generic (and thus less specific) categories, potentially leading to a loss of granularity in our review. This limitation highlights the need for future research studies to adopt standardized terminologies and clear definitions (e.g. as proposed by the classification committee of the Bárány Society) [52]. Secondly, those scales which report on oscillopsia were implemented either in a limited number of studies or in some cases, only single studies. Thirdly, study sample sizes were not infrequently limited and often of mixed etiologies and the value of the PROM used – as indicated by the authors, reflects their judgement. Fourthly, while we assessed the impact of specific scores in distinguishing patients from controls, or between different patient groups (especially in CA patients), the important question of which instrument’s items contributed most to the ability to separate patients from controls was absent and so, the suitability of individual items to the construction of a future tool remains opaque.

Thus, while this systematic review has shed light on the utilization of PRO tools in dizzy and/or ataxic patients, the limitations of the study highlight the need for future research into the development of more suitable tools. This will enhance the validity and reliability of future clinical and research endeavors and addresses the unmet need for PROs.

## Conclusions

This work found that a significant proportion of studies utilize the DHI, despite its limitations, in assessing dizziness. In contrast, only a limited number of studies were identified in which the impact of PROs other than dizziness (e.g., oscillopsia and gait unsteadiness), and commonly experienced by patients with CA, on QoL. Traditionally employed scales in CA such as the SARA, ICARS or the BARS either do not address oculomotor symptoms (SARA), or do not address their impact on QoL (BARS, ICARS, SODA). Therefore, we recommend the development of a novel comprehensive tool for assessing the impact on QoL of oculomotor and mobility limitations on individuals affected by CA. Thereby the recently developed and validated PROM of ataxia could serve as a starting point [23].

### Electronic Supplementary Material

Below is the link to the electronic supplementary material.

Supplementary Material 1

## Data Availability

The data that support the findings of this study are available from the corresponding author upon reasonable request.
