# Supplementary material for: Patient-Related Outcome Measures for Oculomotor Symptoms in the Cerebellar Ataxias: Insights from Non-Cerebellar Disorders
Source: Cerebellum. 2024 Jan 12;23(4):1435–48. doi: 10.1007/s12311-024-01656-3 (PMC11269357; doi:10.1007/s12311-024-01656-3)
Supplement: Supplementary file 1 — Supplementary Material 1 [file 12311_2024_1656_MOESM1_ESM.docx]

## Appendix: Electronic search strategy, coding-scheme for the systematic review and data analysis

**The search strategy was designed by a clinical investigator with relevant domain expertise in neurology (AAT).**

**We searched MEDLINE and Embase for English-language articles, using the following strategies with the following components: (1) defining the clinical syndrome (i.e., ataxia, vertigo, dizziness), (2)** aspects of quality of life investigated **and (3) patient centered complaints investigated focusing on oculomotor and / or vestibular domains. We did not expressly search for hereditary ataxia syndromes, as this was found to result in omitting most relevant studies due to lacking referral to the genetic background. We also performed a manual search of reference lists from eligible articles, and contacted corresponding authors where necessary. We did not seek to identify research abstracts from meeting proceedings or unpublished studies.**

MEDLINE Search *(accessed via PubMed at* [*www.ncbi.nlm.nih.gov/pubmed*](http://www.ncbi.nlm.nih.gov/pubmed)*) on September 20^th^ 2022), number of citations identified: 3525*

(ataxia OR ataxic OR (gait AND (impairment OR imbalance)) OR vertigo OR dizziness OR dizzy OR vestibular OR cerebellar OR cerebellum) AND ((eye movements) OR (ocular motor) OR oculomotor OR vestibular OR saccade OR (smooth pursuit) OR (vestibulo-ocular reflex) OR VOR OR optokinetic OR nystagmus OR gaze OR head impulse OR vision) AND (quality of life OR interview OR questionnaire OR patient-reported OR self-reported OR activities of daily living OR real-world function) NOT (animals[mh] NOT humans[mh]) AND eng[la] NOT review[pt]

Embase search (performed on September 20^th^ 2022) number of citations identified:1766

(ataxia:ab,ti OR ataxic:ab,ti OR (gait AND (impairment OR imbalance)) OR vertigo:ab,ti OR dizziness:ab,ti OR dizzy:ab,ti OR vestibular:ab,ti OR cerebellar:ab,ti OR cerebellum:ab,ti) AND ((‘eye movements’:ab,ti) OR (‘ocular motor’:ab,ti) OR oculomotor:ab,ti OR vestibular:ab,ti OR saccade:ab,ti OR (‘smooth pursuit’:ab,ti) OR (‘vestibulo-ocular reflex’:ab,ti) OR VOR:ab,ti OR optokinetic:ab,ti OR nystagmus:ab,ti OR gaze:ab,ti OR ‘head impulse’:ab,ti OR vision:ab,ti) AND (‘quality of life’:ab,ti OR interview:ab,ti OR questionnaire:ab,ti OR ‘patient-reported’:ab,ti OR ‘self-reported’:ab,ti OR ‘activities of daily living’:ab,ti OR ‘real-world function’:ab,ti) AND [humans]/lim AND [English]/lim AND ([article]/lim OR [article in press]/lim)

Search Results

Our search identified 3671 unique citations, of which 2577 (70.2%) were excluded at the abstract level (Figure 1, main manuscript). A record was excluded only if two scorers (CM, AAT) recommended exclusion (detailed list of predefined reasons for exclusion shown below). We did not demand concordance on reason for abstract exclusion, but, among those abstracts with concordant reasons for exclusion (37.4%, n=1373), the distribution was as follows: **79.3% were not about quality of life or patient-reported complaints;** 14.1% had no data on human subjects with vertigo, dizziness or ataxia**; 4.7% had no original data; 1.7% were not reporting on the assessment of oculomotor /vestibular features and 0.1% were not in English.**

We further examine 1094 full manuscripts. After initial screening, there were a total of 57 disagreements about study inclusion for the two reviewers (RG and AAT, kappa=0.89). These differences were resolved by discussion. Overall agreement on reason for exclusion was 58.9%. We demanded concordance on reason for full-text exclusion and resolved differences by discussion.

At the end of our full-text review, 627 were excluded and 467 were considered eligible (Figure 1, main manuscript). These eligible studies represented 12.7% of the total (n=3671). Among all full-text manuscripts excluded (17.1%), the distribution of reason for exclusion was as follows: **60.1% were not about quality of life or patient-reported complaints;** 12.3% had no data on human subjects with vertigo, dizziness or ataxia**; 6.9% had no original data; 17.2% were not reporting on the assessment of oculomotor /vestibular features; 2.6% no fulltext manuscript could be retrieved, and 1.0% were not in English.**

### Coding schema for abstract and full-text reviews

**All gathered literature was subject to title/abstract screening by two independent reviewers (RG and AAT). Full-text screening was then applied to all citations considered eligible or possibly eligible by at least one reviewer. Two independent reviewers (RG and AAT) determined whether full-text manuscripts were eligible and, if not, provided a reason for exclusion. Differences were resolved by discussion and consensus. AAT completed a hand search of the reference lists of selected articles for additional citations. For citations identified by hand search, the full process was repeated iteratively until no additional manuscripts were found for inclusion. We calculated inter-rater agreement on full-text inclusion using Cohen’s kappa.(Cohen, 1960) A formal review protocol was not registered or posted.**

Abstract Review Coding Rules

1) Coding status options are “Yes”, “No”, “Maybe”. We will review full text of “Yes” and “Maybe”. The purpose of “Yes” vs. “Maybe” is to look at kappa values agreement on “Yes” vs. “Maybe”.

2) Err on the side of “Maybe” if there is doubt about a “No”; this is more conservative.

3) If there is only a title, exclude it only if you feel confident; otherwise code it as “Maybe”.

4) Each "No" or "Maybe" should be coded with a reason for exclusion.

5) Reasons for exclusion are listed below 0-5. Go through them in order from 0 to 5 for each abstract, coding the first reason for exclusion only, not multiple reasons for exclusion. Only code "0" for “not English” if you are sure it is “not English”.

6) Two independent raters will code reason for exclusion, but we will not mandate agreement on exclusion reason at the abstract level.

7) Occasionally an abstract seems inappropriate for another reason. In such cases, code as “other”. There should be few “other” codings.

Abstract Reasons for Exclusion

| 0 | not English | manuscript is not in English |
| --- | --- | --- |
| 1 | no data | review paper; no original patient data |
| 2 | not ataxia / vertigo / dizziness | no data on human subjects with ataxia, vertigo or dizziness |
| 3 | Not about quality of life | not about quality of life or patient-reported complaints |
| 4 | not vestibular / oculomotor | not reporting on vestibular / oculomotor domains |
| 5 | other | any other reason abstract is not included |

Full-Text Review Coding Rules

1) Coding status options are “Yes” or “No”.

2) Each “No” should be coded with a reason for exclusion.

3) Reasons for exclusion are listed below 0-5. Go through them in order from 0 to 5 for each full text, coding the first reason for exclusion only, not multiple reasons for exclusion.

4) Two independent raters will code reason for exclusion, and we will mandate agreement on exclusion reason at the full text level.

5) Coding differences will be adjudicated or consensus will be developed through dialogue.

Full-Text Reasons for Exclusion

| 0 | No full-text manuscript available | No access to full-text manuscript. |
| --- | --- | --- |
| 1 | not English | manuscript is not in English |
| 2 | no data | review paper; no original patient data |
| 3 | not ataxia / vertigo / dizziness | no data on human subjects with ataxia, vertigo or dizziness |
| 4 | Not about quality of life | not about quality of life or patient-reported complaints |
| 5 | not vestibular / oculomotor | not reporting on vestibular / oculomotor domains |

**References**

Cohen, J. (1960). A coefficient for agreement for nominal scales. *Educ Psychol Meas* 20**,** 37-46.
